# The Early Youth Engagement in first episode psychosis (EYE-2) study: pragmatic cluster randomised controlled trial of implementation, effectiveness and cost-effectiveness of a team-based motivational engagement intervention to improve engagement

**DOI:** 10.1186/s13063-021-05105-y

**Published:** 2021-04-12

**Authors:** Kathryn Greenwood, Rebecca Webb, Jenny Gu, David Fowler, Richard de Visser, Stephen Bremner, Iga Abramowicz, Nicky Perry, Stuart Clark, Anastacia O’Donnell, Dan Charlton, Rebecca Jarvis, Philippa Garety, Sunil Nandha, Belinda Lennox, Louise Johns, Shanaya Rathod, Peter Phiri, Paul French, Heather Law, Jo Hodgekins, Michelle Painter, Cate Treise, James Plaistow, Francis Irwin, Rose Thompson, Tanya Mackay, Carl R. May, Andy Healey, Richard Hooper, Emmanuelle Peters

**Affiliations:** 1 R&D, Sussex Partnership NHS Foundation Trust, Hove, UK; 2 School of Psychology, University of Sussex, Falmer, UK; 3 University of East Anglia, Norwich, UK; 4 Brighton and Sussex Medical School, University of Sussex, Falmer, UK; 5 Brighton and Sussex University Hospitals NHS Trust, Brighton, UK; 6 Sussex Partnership NHS Foundation Trust, Hove, UK; 7 King’s College London, Institute of Psychiatry Psychology and Neuroscience, London, UK; 8 South London and Maudsley NHS Foundation Trust, London, UK; 9 Department of Psychiatry, University of Oxford, Oxford, UK; 10 Oxford Health NHS Foundation Trust, Oxford, UK; 11 Southern Health NHS Foundation Trust, Southampton, UK; 12 Pennine Care NHS Foundation Trust, Ashton-under-Lyne, UK; 13 Manchester Metropolitan University, Manchester, UK; 14 Greater Manchester Mental Health NHS Foundation Trust, Greater Manchester, UK; 15 Cambridge and Peterborough NHS Foundation Trust, Cambridge, UK; 16 Norfolk and Suffolk NHS Foundation Trust, Norwich, UK; 17 McPin Foundation, London, UK; 18 Faculty of Public Health and Policy, London School of Hygiene and Tropical Medicine, London, UK; 19 Institute of Population Health Sciences, Queen Mary University of London, London, UK

**Keywords:** Psychosis, Early intervention, Engagement, Intervention, RCT, Economic evaluation, Process evaluation

## Abstract

**Background:**

Early Intervention in Psychosis (EIP) services improve health outcomes for young people with psychosis in the medium–long term, but 25% of young people disengage in the first 12 months with costs to their mental health, families, society and the NHS. This study will evaluate the effectiveness, cost-effectiveness and implementation of a team-based motivational Early Youth Engagement (EYE-2) intervention.

**Method:**

The study design is a cluster randomised controlled trial (RCT) with economic evaluation, comparing the EYE-2 intervention + standardised EIP service to standardised EIP service alone, with randomisation at the team level. A process evaluation will evaluate the delivery of the intervention qualitatively and quantitatively across contexts.

The setting is 20 EIP teams in 5 sites: Manchester, South London, East Anglia, Thames Valley and Hampshire. Participants are young people (14–35 years) with first episode psychosis, and EIP staff.

The intervention is the team-based motivational engagement (EYE-2) intervention, delivered alongside standardised EIP services, and supported by additional training, website, booklets and social groups. The comparator is the standardised EIP service. Both interventions are delivered by EIP clinicians.

The primary outcome is time to disengagement (time in days from date of allocation to care coordinator to date of last contact following refusal to engage with EIP service, or lack of response to EIP contact for a consecutive 3-month period). Secondary outcomes include mental and physical health, deaths, social and occupational function, recovery, satisfaction and service use at 6, 12, 18 and 24 months.

A 12-month within-trial economic evaluation will investigate cost-effectiveness from a societal perspective and from an NHS perspective.

**Discussion:**

The trial will provide the first test of an engagement intervention in standardised care, with the potential for significant impact on the mental health and wellbeing of young people and their families, and economic benefits for services. The intervention will be highly scalable, supported by the toolkit including manuals, commissioning guide, training and resources, adapted to meet the needs of the diverse EIP population, and based on an in-depth process evaluation.

**Trial registration:**

ISRCTN 51629746 prospectively registered 7th May 2019. Date assigned 10th May 2019.

**Supplementary Information:**

The online version contains supplementary material available at 10.1186/s13063-021-05105-y.

## Administrative information

**Table:** 

Title {1}	The Early Youth Engagement in first episode psychosis (EYE-2) study: pragmatic cluster randomised controlled trial of implementation, effectiveness and cost-effectiveness of a team-based motivational engagement intervention to improve engagement
Trial registration {2a and 2b}.	**Trial registration:** ISRCTN: 51629746 Prospectively registered 7th May 2019
Protocol version {3}	4: 3rd April 2020
Funding {4}	This study was funded by the UK National Institute for Health Research through its Health Service & Delivery Research scheme (grant number 16/31/87). The funder had no input into the study design, the collection, management, analysis or interpretation of the data, the writing of the report, or the decision to submit the report for publication.
Author details {5a}	^*1*^ *R&D, Sussex Partnership NHS Foundation Trust, Hove, UK.* ^*2*^ *School of Psychology, University of Sussex, Falmer, UK.* ^*3*^ *University of East Anglia, Norwich, UK.* ^*4*^ *Brighton and Sussex Medical School, Falmer UK* ^*5*^ *Brighton and Sussex University HospitalsNHS Trust, Brighton, UK* ^*6*^ *Sussex Partnership NHS Foundation Trust, Hove, UK.* ^*7*^ *King’s College London, Institute of Psychiatry Psychology and Neuroscience, London, UK.* ^*8*^ *South London and Maudsley NHS Foundation Trust, London, UK.* ^*9*^ *Department of Psychiatry, University of Oxford, Oxford, UK.* ^*10*^ *Oxford Health NHS Foundation Trust, Oxford, UK.* ^*11*^ *Southern Health NHS Foundation Trust, Southampton, UK.* ^*12*^ *Pennine Care NHS Foundation Trust, Ashton-under-Lyne, UK* ^*13*^ *Manchester Metropolitan University, Manchester, UK* ^*14*^ *Greater Manchester Mental Health NHS Foundation Trust, Greater Manchester, UK.* ^*15*^ *Cambridge and Peterborough NHS Foundation Trust, Cambridge, UK* ^*16*^ *Norfolk and Suffolk NHS Foundation Trust, Norwich UK.* ^*17*^ *McPin Foundation, London, UK.* ^*18*^ *Faculty of Public Health and Policy, London School of Hygiene and Tropical Medicine, London UK.* ^*19*^ *Institute of Population Health Sciences, Queen Mary University of London, UK.*
Name and contact information for the trial sponsor {5b}	Sussex Partnership NHS Foundation Trust. Research & Development Department, Sussex Education Centre Nevill Avenue, Hove, BN3 7HZ. United Kingdom + 44 (0)300 304 0088 researchgovernance@sussexpartnership.nhs.uk
Role of sponsor {5c}	In line with the UK policy framework for health and social care research, the sponsor took on overall responsibility for effective arrangements to be in place to set up and run the trial and report its findings

## Introduction

### Background and rationale {6a}

In England, 1–2% of the population [1] or 7500 new young people each year [2] develop psychosis. Psychosis can have devastating consequences, with significantly poorer quality of life and high disability adjusted life year losses [3]. People with psychosis die up to 25 years earlier than the general population [4], one third from suicide, usually within the first 3–5 years from diagnosis [5, 6]. The first 2–3 years are pivotal in determining long-term trajectories [7–12]. Early Intervention in Psychosis (EIP) services are pro-active, person-centred mental health services offering early detection and treatment in this critical 3-year period [2, 13–24]. The recent Access and Waiting Time Standards [25], published in 2016 by NHS England, require that Clinical Commissioning Groups offer a National Institute for Health and Care Excellence (NICE) concordant EIP service within 2 weeks from referral for all new emerging psychosis cases in England.

Despite these guidelines, treatment disengagement from services is high [26–33]: estimated at 30% of young people in a recent systematic review across all service types and follow-up periods [26], and 25% within the first 12 months in standalone EIP services [27, 28], including in our own pilot study [34–36]. This is a significant problem. National policy, investment and service structure are focused on ensuring that young people are proactively engaged in assessment and offered a full EIP care package to prevent them ‘falling through the gaps’, receiving inadequate care, poor outcomes and greater subsequent healthcare use [25], but 1 in 4 disengage. There is limited evidence for methods to promote engagement in the subsequent 3 years. Our work has begun to provide this evidence [34–37]. We now understand why people disengage and are testing the effectiveness and cost-effectiveness of a team-based motivational engagement intervention to reduce disengagement from EIP services.

There is sustained interest in increasing access to EIP services for people of all ages who develop a first episode of psychosis, and a clear need to prevent disengagement. Engagement with EIP services leads to increased service user satisfaction, fewer symptoms, relapses and hospital admissions, better health, wellbeing, social and occupational function and fewer suicides [12, 38–41] in the medium to long term [17–20, 38]. Disengagement of young people with psychosis represents a significant cost to their health and wellbeing and impacts on families, society and the NHS. There is an expressed need from researchers and NHS management to focus on engagement, with some researchers suggesting it is the most important outcome of EIP services [42]. The College Centre for Quality Improvement has made time to disengagement a recent EIP audit requirement [43]. Furthermore, Access and Waiting Time Standards are supported by NHS England, who are committed to further access and engagement targets up to 2020 [25]. EIP service access is ‘a clear national priority for the NHS’, and local NHS services must include EIP development in their immediate and long-term sustainability and transformation plans [25]. This commitment has been supported by £70 million for staff and training to 2020 [44]. Yet disengagement from these services threatens the quality of health outcomes and nullifies this investment for 25% of young people. The financial cost of psychosis to society, including healthcare, families, unemployment and death, is estimated at £11.8 billion per year [45]. EIP services demonstrate savings of 30–50% over standard care, over periods of at least 8 years [46, 47]; £5000 per person per year based on days in hospital [37]; £7972 net savings per person after 4 years, £6870 in the next 4–10 years and £15 for every £1 spent on EIP services after 10 years [48]. Even with suboptimal engagement, EIP is estimated to result in £63 million of savings per year to society, £34 million of these to the NHS [49].

Our initial Early Youth Engagement (EYE) project [34–36] developed a team-based motivational engagement intervention, drawing on views of service users and their families of barriers and facilitators to engagement, and on literature that disengagement is linked to younger age, substance use, coping styles, family contact and knowledge of services [26, 29, 31]. To date, limited evidence from our own work identifies strategies to maintain engagement from initial assessment or when a young person begins to disengage. Our Delphi consultation with clinicians and managers reached consensus on the EYE intervention and resources that were both important and feasible to deliver, based on the views of young people and their families. Our pilot study found that service disengagement decreased from 24% prior- to 14.5% post-EYE intervention. Qualitative data from service users, families and staff revealed improvements in personal recovery (social inclusion, hope, trust, practical goals) and engagement (communication, collaboration, family involvement). Families reported feeling more reassured that they knew how to support their young person, and staff felt more pride and professionalism in their service due to having access to high-quality resources and information.

However, the original EYE pilot study was conducted on only 298 service users, and whilst it showed promising results in reducing disengagement, the pre- and post-intervention comparison methodology was not designed to formally assess the effectiveness of the intervention. There was no evaluation of the cost-effectiveness of the intervention and limited evaluation of implementation.

The current protocol presents the methodology of a pragmatic cluster randomised controlled trial (RCT) to investigate the effectiveness, cost-effectiveness and implementation of the team-based motivational engagement intervention aimed at reducing disengagement from EIP services—the EYE-2 project.

### Objectives {7}

#### Aims

The main research aims are:

(i) To evaluate the effectiveness of the team-based motivational engagement EYE-2 intervention with respect to the primary outcome, time to disengagement and secondary outcomes: mental and physical health, deaths (including suicide), social and occupational function, recovery, satisfaction and service use derived from routine service data (Health of the Nation outcome Scale (HoNOS), Process of Recovery Questionnaire (QPR), DIALOG questionnaire [50–52] at 0, 6, 12, 18 and 24 months.
(ii) To determine societal and NHS costs, cumulative cost savings and overall cost-effectiveness of improved EIP engagement and produce a commissioning guide, with GP commissioner input.
(iii) To develop and test a framework for implementation through a large-scale process evaluation using (i) Normalisation Process theory (NPT) [53] and (ii) logic models [54], incorporating all clinicians involved in EYE-2 intervention delivery, assessed through questionnaires and qualitative interviews at the start, middle and end of the trial.

#### Hypotheses

The primary hypothesis is that, compared to standard EIP alone, the EYE-2 intervention will increase time to disengagement;

The secondary hypotheses are that, compared to Standard EIP alone, the EYE-2 intervention will:

(i) Improve mental and health outcomes;
(ii) Improve recovery, social and occupational function, and satisfaction;
(iii) Be cost-effective with potential societal and NHS cost-savings.
(iv) Be moderated by effective implementation as measured by the process evaluation questionnaires

### Trial design {8}

**Fig. 1:**
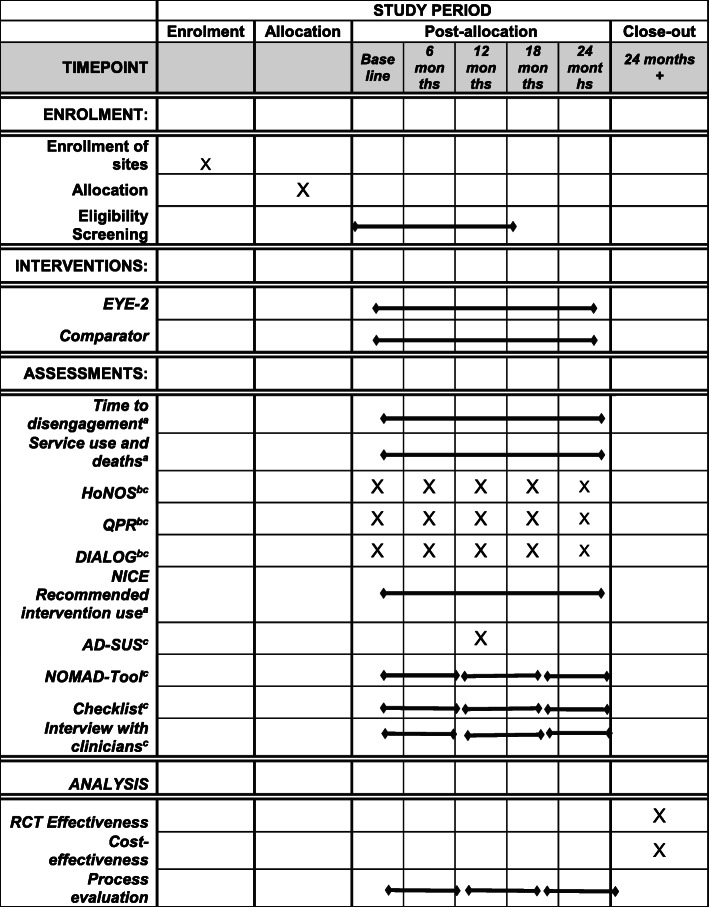
SPIRIT figure. ^a^Routinely recorded by service and collated by RAs in teams. ^b^Routinely collected by clinicians and collated by RAs in teams. ^c^Collected by RAs for AD-SUS, people who disengage, and missing data

Figure 1 comprises the SPIRIT (Standard Protocol Items: Recommendations for Interventional Trials) figure [55].

The trial is a parallel-group cluster RCT, with 1:1 allocation by cluster, stratified by site, to test the effectiveness and cost-effectiveness of the EYE-2 intervention in reducing disengagement compared to standardised standalone EIP service.

Our success (go) criteria are

1. End of month 9—20 teams randomised
2. End of month 16—50% of participants identified
3. End of month 16—Initial fidelity data available at all sites

Our stop criteria at which point the trial will be considered infeasible will be if any of the following apply:

1. End of month 12—< 17 (80% of teams) teams randomised
2. End of month 19—< 40% of participants identified
3. End of month 19—Initial fidelity data available for fewer than 3 sites

The cost-effectiveness study will comprise a 12-month economic evaluation of the EYE-2 intervention undertaken primarily from a societal perspective, accounting for cost impacts within and beyond the mental health sector, with a secondary cost-effectiveness analysis taking a narrower NHS perspective, focusing principally on NHS mental health service utilisation. The economic evaluation will include consideration of mental health outcomes (measured through HoNOS) and improved levels of engagement with EIP services. An alternative 24-month point will be considered however, if the informed consent process leads to withdrawals.

A prospective mixed methods process evaluation will investigate the delivery of the intervention. The process evaluation will be longitudinal (over 2 years) and will be informed by Normalisation Process Theory [53, 56–58] and trial-specific logic models [54].

## Methods: participants, interventions and outcomes

### Study setting {9}

The setting will be 20 UK EIP community teams in South London; Manchester; Hampshire; Thames Valley and East Anglia, derived from 9 NHS trusts across England. Specific trust information is listed in the ISRCTN trial registration.

### Eligibility criteria {10}

All services meet the following specific inclusion criteria: (i) standalone EIP site with at least 2 discrete services; (ii) willingness and capacity for involvement as agreed by clinical services; (iii) identified site principal investigator with academic track record in leading RCTs in psychosis; (iv) regional EIP support; (v) individual service size of at least 35–40 new clearly defined first episode cases per year aged 14–35; (vi) currently capturing NHS England mandated routine outcome data; (vii) systems (IT and staff) in place to increase routine outcome data capture; (viii) geographical spread to include urban and rural locations, ethnic minority variations, and North and South of England.

Service user inclusion criteria are as follows: (i) consecutive referrals to the EIP team during the study recruitment period (ii) aged 14–35; (iii) meeting criteria for a first episode of psychosis (FEP): (F20-F29; F31 ICD-10) [59] as determined by each local service according to their own established criteria. The inclusion criteria used to make these decisions will be recorded for each service and reported for subsequent inspection. Exclusion criteria are (i) a sub-threshold ‘at risk mental state’, not meeting FEP criteria, (ii) referral over the age of 35, (iii) referrals where there is remaining diagnostic uncertainty about psychosis at 12 months and (iv) service exclusion criteria such as organic or intoxication induced psychosis and specific exclusions.

Clinician inclusion criteria for the process evaluation are all clinicians delivering EYE-2 and/or EIP services as part of the RCT. Exclusion criteria are EIP clinicians who are in the EYE-2 group but did not receive training in the intervention and EIP clinicians across both groups who did not provide consent.

### Who will take informed consent? {26a}

This study presents unique challenges as the primary ‘disengagement’ outcome has the potential to impact disproportionately on data completeness in those who are disengaging, who are the least likely to consent. A standard approach of receiving individual informed consent and providing commensurate reimbursement for time is likely to impact on robust data completion. As a result, this intervention will be primarily evaluated using routinely available and routinely collected service data. Data will be collated by the EIP Research Assistant (RA) who will work as part of the clinical team and will transfer anonymised service data to the research team. The study will be widely publicised within each team to all those who are eligible and defined as part of the research cohort. They will receive a study information leaflet, contact details for the trial, promotional material and a pack of local support service information, as recommended by our Patient and Public Involvement (PPI) group. Any service user who requests that their data are not used will be withdrawn from the trial. All remaining service user data will be included.

Service users will be contacted directly for the purpose of collecting a small amount of additional questionnaire data, as part of a research process in which informed consent will be taken, either (i) if a service user has disengaged completely and routine service data are not available or (ii) at the 12-month assessment when non-routine health economic data are collected. In these cases, a member of the clinical team or the clinical RA will make the first contact. All service users who are willing will then be contacted by the study RA who will take informed consent for completion of questionnaire data and will provide £20 remuneration for their time.

For the process evaluation, all EIP clinicians will be invited to take part by the clinical RA. They will be provided with an information and consent sheet in advance and will provide written informed consent prior to the process evaluation.

### Additional consent provisions for collection and use of participant data and biological specimens {26b}

Not applicable. This trial does not involve collecting biological specimens for storage. There are no ancillary studies that require additional consent provisions.

### Interventions

#### Explanation for the choice of comparators {6b}

The current EIP care pathway is variable nationally in adherence to the EIP model, with standalone, hub and spoke services, and specialist workers in community teams. Standalone services that adhere to the EIP model have the best outcomes [60, 61], and recent investment and targets [25] mean many services are moving to this model. All services involved in the current study are standardised in that they are standalone services, adherent to the EIP model core principles of (i) early detection, (ii) assertive engagement, (iii) person and recovery focus, (iv) family focus, (v) work with diagnostic uncertainty, (vi) positive risk-taking and (vii) provision of NICE-recommended interventions [22, 61].

We have selected the standardised EIP pathway as the comparison condition because it is the nationally recommended care pathway for people in England who develop a first episode of severe mental illness (psychosis). Delivery of this pathway and routine outcome measures (HoNOS; QPR; DIALOG) [50–52] are mandated by NHS England [25]. The EIP service model and suite of NICE-recommended interventions are clearly defined. Training and monitoring will ensure that administration and recording of measures and intervention provision is undertaken in the EIP pathway in a robust, standardised way across sites.

#### Intervention description {11a}

Whilst the EIP model outlines what should be done, the EYE-2 intervention is complementary to this pathway, providing detail regarding how staff and teams should operate, and the tools, resources and breadth of social network with which they should work to promote better engagement.

**Fig. 2:**
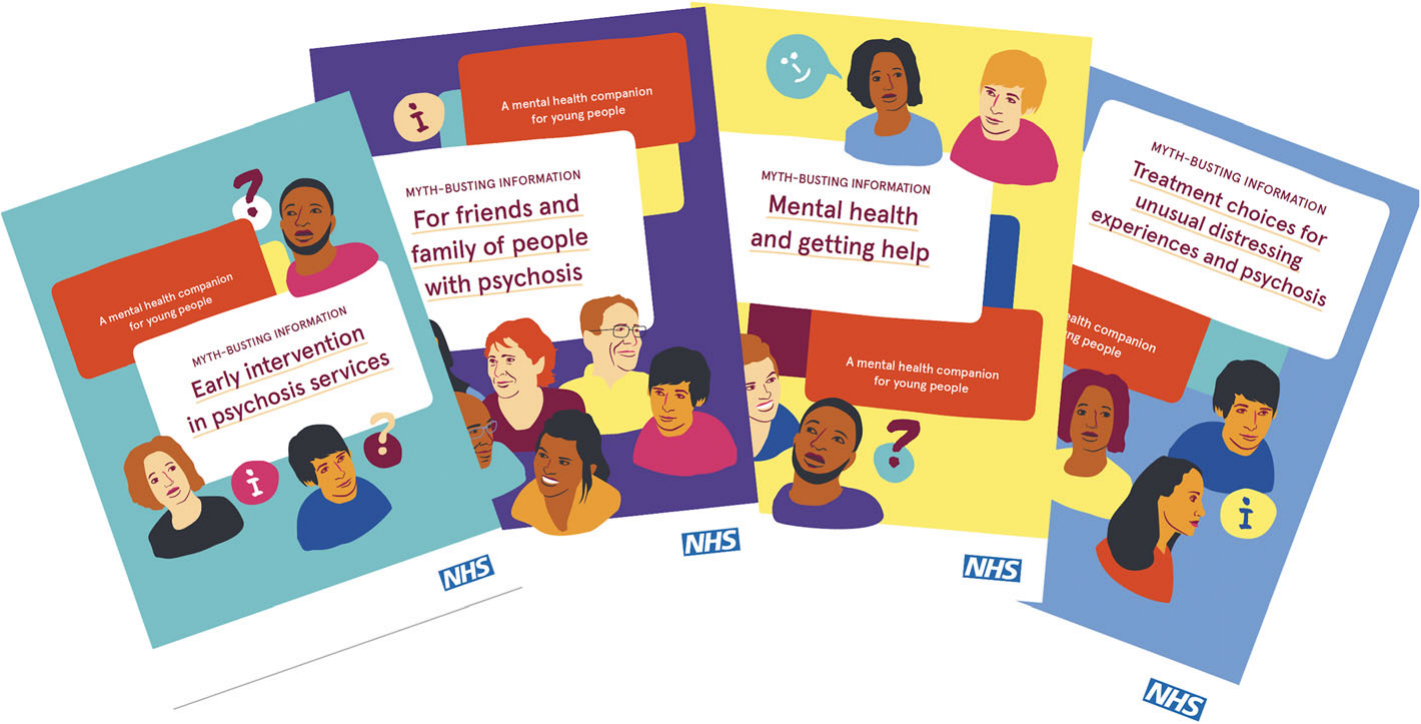
EYE-2 intervention booklets for service users

**Fig. 3:**
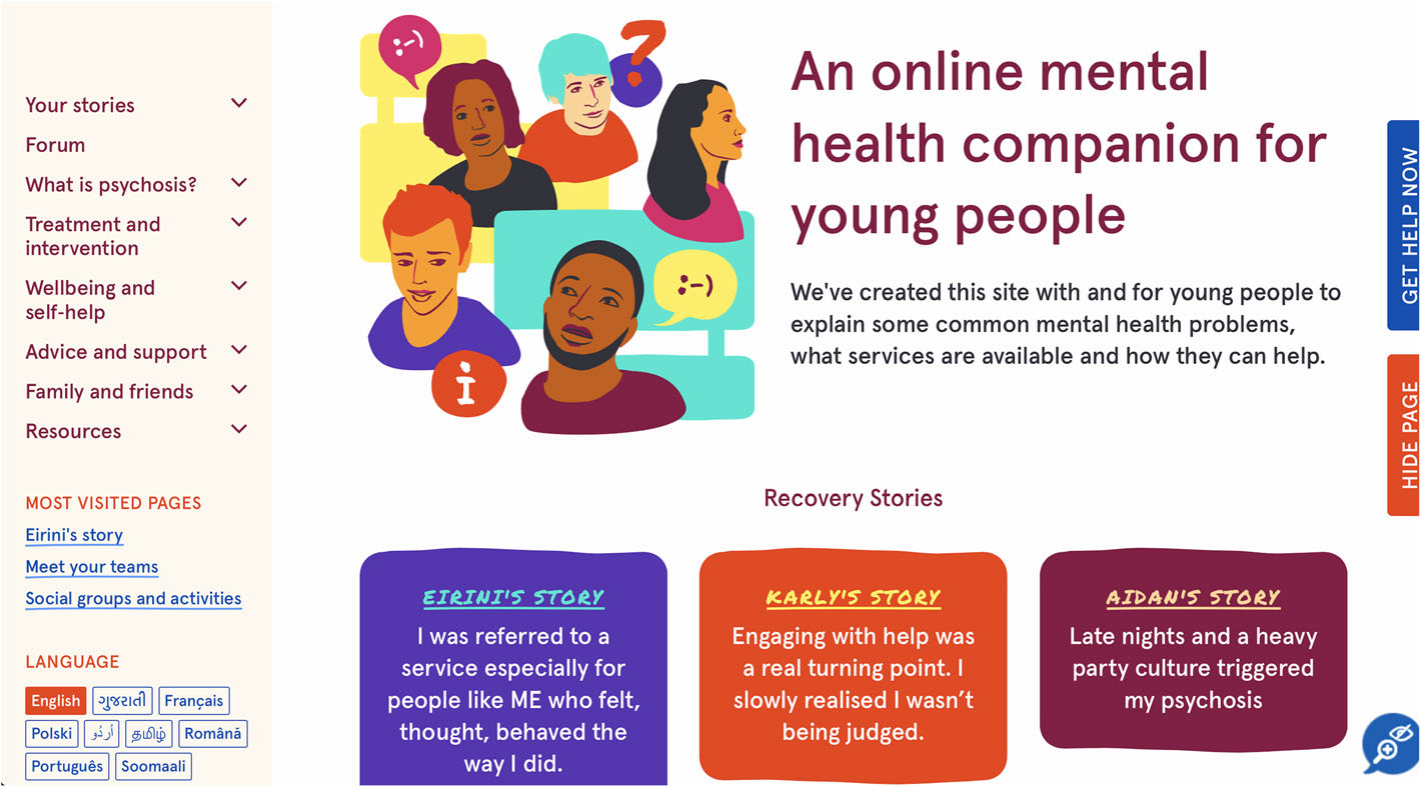
Homepage of the EYE-2 website for service users

The team-based motivational engagement (EYE-2) intervention is delivered by EIP clinicians during their normal routine contacts throughout the entirety of the trial period, supported by training, an intervention manual, booklet series (Fig. 2), website (Fig. 3), friends and family involvement and social groups protocol. It is incorporated into standard EIP teams.

The implementation tool kit is a set of resources provided to each clinician as part of the training. It comprises (i) the implementation manual; (ii) the booklets (mental health and help-seeking, EIP, for friends and family, treatment choices, (iii) the EYE-2 team and individual implementation checklists and (iv) the links to the website and training videos.

The EYE-2 intervention is framed around a novel therapeutic engagement model derived from a previous study (The EYE project) [34–36] and a subsequent implementation study. It is based on motivational interviewing and open social communication and includes the following approaches and resources to support young people to reach their life and treatment goals:

1. Communication: transparent, open and honest communication

All staff are trained by the EYE-2 team in open motivational communication approaches, supported by the website and myth-busting booklet series, which address young people’s real concerns in a direct, honest manner.

2. Social involvement: support of the whole social network

Staff and service users are encouraged to draw on a wide social network of friends, family and peers, in pursuit of the young person’s goals, supported by the friends and family booklet and service user-led social groups run by the PPI lead, and RA, that focus on young people’s own goals and interests and encourage young people to support each other. Training is provided in carers’ rights, and in processes for involving friends and family.

3. Mental health service: collaboration and choice regarding difficult treatment issues

Collaboration and choice are supported by the staff training, and service user-led training videos, shown during the staff training, and available on the EYE-2 website regarding difficult treatment issues, risk and hospital admission, which ordinarily impact on engagement. It is supported by the ‘challenges you may face’ section on treatment in the Family and Friends booklet and by the ‘Treatment choices booklet’, a comprehensive, highly valued, user-friendly, honest review of treatment options, co-produced with service users, carers, and all clinical disciplines.

4. Mental health staff: hopeful support for meaningful goals and needs

The staff training, based on motivational interviewing and open social communication, is supported by service user-led training videos and promotes a hopeful, motivational, goals-focussed approach. Website resources will enable staff to be knowledgeable, with information at their fingertips to support their service users.

5. Addressing personal barriers

Personal barriers to engagement are addressed by reaching out to service users through the discussion forum on the website, the ‘addressing personal barriers to talking’ sections in the booklets, and the social groups that are attended and co-led by service users.

#### Criteria for discontinuing or modifying allocated interventions {11b}

Consistent with the engagement approach, a service user and clinician work together such that the intervention delivery is tailored to the needs and preferences of the service user. Any concerns over safety will be identified through adverse reactions (ARs) and serious adverse reactions (SARs), and modification to the overall intervention will in the first instance occur through protocol amendments.

#### Strategies to improve adherence to interventions {11c}

Adherence to the intervention will be supported by the implementation toolkit (manual, resources and EYE-2 checklist), initial and booster training programme, and large-scale process evaluation, and by additional resources and activities that are requested to support implementation throughout the trial.

##### EYE-2 training programme

The EYE-2 training is delivered by the chief investigator and research fellow, supported by the site PPI team (PPI lead, 1–2 local service users, and 1 local carer who will provide a local perspective on service provision and the EYE-2 approach), and practically by the EIP RA. The training will last 1.5 days. Core sessions include (i) introduction to the EYE-2 intervention and resources, (ii) the value of hopeful care coordination, (iii) goal-focussed care planning, (iv) service user-led introduction to honest open communication, (v) carers rights and protocol for engaging family and friends, (vi) peer workers and social groups, (vii) motivational interviewing for goal-focussed engagement, (viii) applying open communication approaches in the context of risk, mental health exacerbations, treatment and admissions, (ix) the implementation process and local implementation plans and (x) the research process—ethics, consent, promotion and awareness raising. Two rounds of each training are offered at each site, at least 1 month apart, to enable staff to attend whilst maintaining service delivery, and to accommodate leave and absence. A preparation and consolidation phase of up to 1 month will allow final preparations for RCT start. Additional booster training will be offered approximately 6-monthly throughout the intervention, with content being informed by feedback collected in earlier training, in the qualitative interviews, and from staff at study sites.

##### Adherence monitoring in the process evaluation

Monitoring of adherence to the intervention will be informed by the process evaluation questionnaire data which will be collected from all consenting clinicians at 3 time points (start, middle, end) during intervention delivery, and from the team clinical RA in each team. The questionnaires will evaluate adherence to the EIP and EYE-2 intervention models, individual and team policies and practices. The EYE-2 questionnaires will be used to describe a threshold for effective implementation based on clinicians’ use of EYE-2 resources with EYE-2 service users. A random sub-sample of 33–40 clinicians across all EYE-2 teams at the same three time points will complete a brief semi-structured interview to explore barriers and facilitators to intervention delivery in more detail in relation to context and turbulence. Baseline and mid-trial fidelity assessments will be summarised and fed back to services to boost fidelity.

##### Contamination protocol

A contamination protocol will be provided to teams to ensure that resources are not shared outside of EYE-2 teams, that staff who move from an EYE-2 team to a standard EIP team do not take resources with them, and wherever possible, that they do not work directly with the study cohort participants from the control group. We will monitor any transfer of EYE-2 resources between study groups, and any relevant training.

#### Relevant concomitant care permitted or prohibited during the trial {11d}

There will be no restrictions to the usual concomitant care and interventions provided during the course of the trial. A case note screen will record NICE guidelines interventions provision.

#### Provisions for post-trial care {30}

Ancillary and post-trial care will be provided by standard NHS EIP services, except for service users who have voluntarily disengaged from services, who will be provided with information on local support services, and appropriate NHS services as required during any research assessment.

### Outcomes {12}

Outcomes will be reported separately for the RCT, health economic evaluation and process evaluation.

#### RCT outcome measurement

The primary outcome for the RCT is time to disengagement [28]. Secondary outcomes will comprise the NHS England mandated routinely collected clinician-rated [50] and patient-reported outcomes [51, 52] at 0, 6 and 12 months (18 and 24 for those identified in the 0–6 months of the trial). These secondary outcomes evaluate behavioural, functional, mental and physical health and social-occupational problems, patient-reported recovery, subjective quality of life and treatment satisfaction. Service use in terms of provision of NICE-recommended interventions, and deaths will also be documented.

#### Health economic evaluation

The primary outcome will be the Adult Service Use Schedule (AD-SUS) [62, 63], administered at 12 months, which will measure, through participant self-report, wider service use over follow-up and employment-related outcomes. To support the economic evaluation, a common NHS England mandated dataset will be used to measure patient outcomes (HoNOS scores) and resource use pertaining to contact with EIP and other interventions developed for this patient group, psychiatric inpatient admissions, service use relating to section 136 and A&E contacts will also be recorded.

#### Process evaluation outcome

Implementation of the intervention will be evaluated longitudinally at 3 time points (early, middle and end of the trial) using both questionnaires (EYE-2 and EIP checklists, and the NOMAD tool, [64] which explores attitudes and behaviours towards implementation-based on normalisation process theory) and qualitative interviews.

### Participant timeline {13}

Figure 1 illustrates the SPIRIT figure for EYE-2.

### Sample size {14}

Time to disengagement will be analysed using frailty analysis to adjust for clustering by service. Simulation confirms that 10 clusters per group (*n* = 950) will achieve 90% power to detect a difference corresponding to 12-month disengagement rates of 25% (standard 12-month disengagement rate from EIP service) [26–28] vs 15%, assuming time to disengagement has an exponential distribution; intracluster correlation of 0.05 [65] drop-out rate of 10% per year; conservative significance level of 3% to correct for inflation of type I error due to small cluster numbers; variable cluster size modelled as a uniform random variable between 35 and 60; recruitment at referral; and 12 months recruitment plus 12 months follow-up. Simulations were conducted using the SimSam package in Stata 14, see details and code at https://github.com/richard-hooper/simsam/tree/EYE2 [66, 67].

### Recruitment {15}

The EIP RA working as part of the EIP clinical team in each service will work with the lead clinicians in the clinical team and with reference to the eligibility criteria to determine the cohort of eligible service users in that service. All consecutively referred service users during the recruitment period, who meet criteria, and do not ask to be withdrawn, will form the cohort in each team. Unclear cases will be discussed and agreement reached with the site principal investigator (PI) and study chief investigator (CI).

For the training and process evaluation questionnaire study, all clinicians will be invited to take part verbally and via an online link in an email. A threshold will be set for sufficient staff consent to warrant service entry into the trial. This threshold will be set to at least 80% of all care coordinators, and the team leader, consenting to take part to further ensure that sufficient staff received training to deliver the intervention. For the qualitative study, 33–40 clinicians will be randomly selected from among those who provide consent, sampled purposively to include all clinicians and managers across all 10 EIP teams, to take part in an individual interview.

## Assignment of interventions: allocation

### Sequence generation {16a}

The statistician at the Brighton and Sussex Clinical Trials Unit (CTU) generated a randomisation list comprising permuted blocks of size 2, stratified by site (Manchester, London, Thames Valley, East Anglia, Hampshire) using a tool provided by Sealed Envelope™ [68], an independent online randomisation service.

### Concealment mechanism {16b}

To achieve statistician blinding and to ensure concealment, this was sent to an independent statistician (AMJ) to combine with the teams list, itself randomly ordered within site, by sorting a random number list, which she uploaded to Sealed Envelope.

### Implementation {16c}

The research fellow requested the password-protected concealed allocations online once all the participating teams at a site had reached the threshold for care coordinator and staff recruitment (≥ 80%) and were ready to start.

## Assignment of interventions: blinding

### Who will be blinded {17a}

All researchers involved in the analysis of trial effectiveness and cost-effectiveness data will be blind to allocation status. This includes members of the Brighton and Sussex CTU, statisticians carrying out the RCT analyses, and health economists conducting the cost-effectiveness analysis. In addition, each site will be allocated a ‘blinded’ RA who will code the primary ‘time to disengagement’ trial outcome measure and collect the primary AD-SUS cost-effectiveness data blind to study group. They will also collect additional 12-month data, and data from participants who disengage, blind to study group. Blinding will be ensured by situating blinded researchers in a separate site or building from remaining trial team members, and by ensuring separate sections of meetings, and separate documents as necessary for blinded and unblinded researchers. We will record when data have been collected blind and unblinded for subsequent inspection. A full statistical analysis plan will be written and independently reviewed prior to final analysis being undertaken by the trial statistician who will be kept blind to which trial group is which. Service users, clinicians, remaining local site teams (EIP RA, PPI lead, principal investigator/site lead), trial manager and chief investigator will not be blind to study allocation.

### Procedure for unblinding if needed {17b}

As noted above, service users, clinicians and local site teams will already be unblinded, so this is not applicable.

## Data collection and management

### Plans for assessment and collection of outcomes {18a}

Data collection forms will be provided on the study website at https://www.sussex.ac.uk/research/projects/eye-2/.

#### Data collection methods

There are 3 main methods of data collection in this study that have been specifically designed to maximise data completeness (*see* Fig. 1). First, primary outcome data are recorded routinely by the service, but collated by the RA working within the EIP service and transferred anonymously to the research team (disengagement and service use data, as well as data on use of NICE interventions). The primary outcome of time to disengagement will be calculated by the blinded RA who will determine date of disengagement (date of last contact with the team) and calculate the number of days from allocation to care coordinator to disengagement using casenote data provided by the EIP RA, who will double check the data using the same process, and any discrepancies will be resolved in discussion with the study team. Second, there are data that are routinely collected by EIP clinicians but collated by the researchers (HoNOS; QPR; DIALOG) and transferred anonymously to the study team. These researchers in their role with the team will also collect additional missing routine data. Finally, where routine data are missing at 12 months, for cost-effectiveness data (AD-SUS), and for routine data from those who disengage, a standard informed consent process will be taken prior to data collection as described above, and data including HoNOS, QPR and DIALOG will be collected by telephone or in person. If the service user prefers to complete the questionnaires by telephone, consent will be noted by the researcher, after which the researcher will conduct the HoNOS using a semi-structured interview developed for this purpose in collaboration with the Patient and Public Involvement team, as well as the QPR, the DIALOG and the AD-SUS at 12 months. If service users prefer to complete the data in person, then written informed consent will be taken, a convenient public space will be agreed to meet to complete the measures, and the same process will be followed. Participants will be reimbursed £20 for their time in cash, or as an Amazon.com voucher. If at any point the participant asks to stop taking part, this will be noted and the interview will be stopped. If the participant asks not to be contacted again regarding the study, this too will be noted, and they will not be contacted again. We will record the nature of data collection (clinician, telephone assessment or case note screen) for all outcomes.

Engagement and service use data will be captured continuously from case notes. Secondary routine clinician-rated (HoNOS) and patient-reported (QPR/DIALOG) questionnaire data will be collected at 0, 6, 12, 18 and 24 months. Baseline will comprise the first − 4 to + 6 weeks to allow for baseline assessments in hospital immediately prior to EIP allocation; follow-up data will be collected at each time point − 2/+ 4 weeks. The study will include a 12-month recruitment period and an additional maximum 12-month follow-up period. Those who enter the study at the start of the cohort will be followed up for 24 months, whilst those who enter at the end of the cohort will be followed up for 12 months.

#### Clinician training in robust data collection

An initial half-day training will be provided for clinicians in both EYE-2 and control teams at each site on robust data collection and recording. This will be supported by a data collection manual and coloured user-friendly questionnaire packs and resources. The training will include (i) team values and data collection, (ii) an introduction to the questionnaires, (iii) identifying and overcoming barriers to data collection, (iv) clinically meaningful use in care planning (v) top tips and resources to support implementation. This training programme has been adopted by NHS England and translated into an e-learning package for all EIP services https://www.e-lfh.org.uk/programmes/early-intervention-in-psychosis/. It is incorporated into current National Clinical Audit of Psychosis outcomes training. The full training package will thus be 1/2 a day on robust data collection training for control sites and a 2-day training to include also the EYE-2 training at intervention sites.

#### Researcher training in data collection and data entry

RAs will be provided with initial training in cultural differences by author SR, based on her cultural adaptation framework with additional information gained from earlier EYE-2 work. The model uses the bio-psycho-spiritual-social model of illness, taking into account the philosophical orientation of the individual, societal factors that impact on experiences, trust and technical adjustments to interventions, including the role of religion and spirituality and concepts such as body and mind, self and other, individual and collective goals [69–72].

Researchers will also be trained in study procedures, data collection and intervention support roles, MACRO data entry procedures and protocols. They will be supervised by the site PIs and research trial manager. Checks will be undertaken of the accuracy of data coding and entry at each site, by the blind RA.

Process evaluation questionnaire data will be collected from all consenting clinicians at 3 time points (start, middle, end) during intervention delivery. The questionnaires will take 20–30 min and can be completed online by email link, or by telephone, or in person, at an NHS base or elsewhere local to the clinician. All clinicians and managers in each intervention team will be invited to complete the process evaluation questionnaires. A random sub-sample of at least 2 clinicians and 1 manager at each of the 10 intervention services (*n* = 33–40) will also complete a brief semi-structured interview to explore barriers and facilitators to intervention delivery including context and turbulence at 3 time points, with at least 10 participants completing each time point of beginning, middle and end of the intervention. Each clinician will be interviewed in a face-to-face or telephone individual interview with a clinical EIP researcher from another site, at a time and place that is convenient to them. The interview will take 20–30 mins and will be audio-taped for subsequent transcription. It will be guided by the process evaluation topic guide. The process evaluation topic guide will be informed by Normalisation Process Theory and the logic model for EYE-2.

#### Description of outcomes and their reliability and validity

##### Primary RCT outcome

The primary outcome is time to disengagement (in days, from date of allocation to care coordinator to date of last contact following either refusal to engage with an EIP team or lack of response to EIP contact for 3 consecutive months). For participants who remain engaged until the end of the study follow-up period, time to disengagement is treated as censored (unknown) beyond this point. This definition is widely used in engagement research [28, 31, 73, 74]. People who engage intermittently every few weeks or via text or phone would still be engaged. Service users who move to a service not in the study, or to the opposite study group, or move out of the UK and cannot be referred to a mental health service will no longer be receiving the intervention are deemed lost to follow-up.

##### Secondary RCT outcomes

1. Health of the Nation Outcome Scale (HoNOS [50];)

The HoNOS is a 12-item clinician-rated scale, which covers key health and social outcomes under four subscales of behavioural (aggression, self-harm, substance use), functional (cognitive and physical), mental health (psychosis, depression, other) and social-occupational problems (relationships, activities of daily living, living conditions and occupation). Each item is rated from 0 (no problem) to 4 (very severe), for the preceding 2 weeks and summed to produce a total and subscale scores. The HoNOS is used to ‘cluster’ mental health service users according to clinical need. These clusters map onto NHS commissioning tariffs and can be used to determine cost-savings. It has good internal consistency and validity and adequate sensitivity to change [75, 76].

2. Process of Recovery Questionnaire (QPR [51];)

The Process of Recovery Questionnaire is a 15-item patient-reported outcome measure, developed by psychosis service users to capture recovery. Items include social inclusion, assertiveness, motivation, positive relationships, purpose, empowerment, self-esteem, self-efficacy, meaningful activity, understanding, acceptance, enjoyment and positive risk-taking, each rated on a 5-point scale from strongly disagree to strongly agree. Internal consistency is 0.89, convergent validity is 0.73, test–retest reliability is 0.74 and sensitivity to change is 0.40 [77].

3. DIALOG [52]

The DIALOG assesses patient-reported satisfaction across two subscales of (i) subjective quality of life including health (mental and physical), function (work, leisure), social (friendships/family relationships), accommodation and personal safety; and (ii) treatment satisfaction (practical and mental health support, medication) all rated on a 7-point scale from Totally Dissatisfied to Totally Satisfied. It has adequate reliability (Cronbach’s alpha = .57–71) and is valid (*r* = 0.95 with Manchester Assessment of Quality of Life) [52].

4. NICE-recommended intervention use

Use of NICE-recommended interventions is recorded on electronic care record systems as SNOMED-CT (Systematized Nomenclature of Medicine Clinical Terms) Terms, a set of comprehensive scientifically validated terms used internationally, and designated for NHS use [26]. Relevant codes for EIP are Cognitive Behaviour Therapy for psychosis, Family Interventions for Psychosis, Antipsychotic medication and monitoring, Physical Health interventions and monitoring, Supported employment and vocational/educational rehabilitation; care and treatment planning, substance use assessment and intervention.

##### Cost-effectiveness outcomes

The primary outcome will be societal service use. NHS service use will be the secondary outcome. The primary endpoint will be at 12 months.

1. Adult service use schedule [62, 63]

The AD-SUS is a structured questionnaire designed to elicit self-reported contact with services and employment outcomes across a wider spectrum of services including primary care, social services, police and criminal justice contacts, education and training services and occupational outcomes. It has been used widely in various forms in economic evaluations of child/adolescent and adult mental health services.

2. Service use and deaths

Service use data, as advised by our GP commissioner, will include (i) number of days spent in hospital; (ii) number of A&E presentations and (iii) number of instances of section 136 use. Deaths including from suicide will also be recorded.

##### Process evaluation outcomes

Primary outcome will be delivery across sites as measured in the EYE-2 group by (1) the EYE-2 checklist, which assesses individual and team adherence to the EIP and EYE-2 model, access to relevant training (motivational interviewing, open dialogue, EIP or engagement), the working alliance inventory and subscales of the spontaneous self-affirmation measure. (2) The NOMAD tool [64] which will explore attitudes and behaviour towards the intervention informed by Normalisation Process Theory and logic models; and in the control group by (1) an adapted EIP checklist, similar to the one above to assess adherence to the EIP model, working alliance, self-affirmation, relevant training, principles of good practice that are promoted in the EYE-2 model, and inadvertent access to EYE-2 intervention resources.

Elements of the Royal College of Psychiatrist EIP self-assessment tool [43] and other national and local service-level data will be collected in both groups to describe service delivery and context.

EIP RAs will also complete a second section of the EYE-2–EIP checklist that relates to broad service profiles and practices that are proposed to influence EYE-2 and EIP delivery. The questionnaire will be completed with reference to team policies and in consultation with the team leader and will be conducted in all teams in both groups of the study.

Secondary outcomes will be the thematic framework derived from the qualitative sub-study that influences delivery, including context and turbulence.

### Plans to promote participant retention and complete follow-up {18b}

Research suggests that follow-up rates are improved by using shorter assessments, reminding service users about subsequent follow-up, updating contact details, providing reimbursement, and utilising RAs to collect additional data [78, 79]. We have incorporated all of these approaches into the current strategy. Patient-reported outcomes are brief (approximately 15 min, 25 min including the Adult Service Use Schedule) and can be completed by telephone. Participants will be advised that they may be contacted and will be reimbursed £20 for their participation. The maximum follow-up rate of 8 questionnaire assessments per RA per week if no data are available routinely is achievable based on previous research in these sites. The use of routine outcome data will reduce the risk of disproportionate data loss for participants who are minimally engaged.

RAs have a range of approaches to enhance completion of secondary routine data collection. The clinical EIP RA will provide reminders to clinicians when routine outcomes are due and overdue. They will provide individual outcome graphs for service users, clinicians and whole team graphs, once follow-up outcomes are available, to promote clinically meaningful use. The research team will provide a monthly report of data completeness and missing data, by team and site, to site PIs, team lead and clinical link person in each team. This will raise awareness regarding follow-up rates and missing data. The clinical EIP RA can also collect missing routine data in person or by telephone on behalf of the team. Participants who disengage will be invited to provide data as part of a research process.

### Data management {19}

#### Data quality

The CTU data manager and trial manager will undertake data quality checks in accordance with the data management plan, supported by close liaison with the site PIs and RAs at each site. The CTU will develop an electronic Case Report Form (eCRF), using Elsevier MACRO™. The system is Good Clinical Practice (GCP) and 21 CFR Part 11 compliant with a full audit trail and database lock functionality. Staff at each site will be trained to ensure that data are captured reliably using a standard eCRF proforma according to the processes defined above. Proforma data will be recorded anonymously using a distinct code to represent each service user, monitored for completeness, collated and entered consistently in anonymised form by the clinical EIP RA in each service on the MACRO database for the CTU and analysis team who will be blind to study group. Limits will be placed on variable ranges in the eCRF to reduce risk of inaccurate data entry. The RA who will also be blind to study group will enter data that is collected following informed consent. The CTU will monitor for data completeness and accuracy on a monthly basis. The statistician will provide regular reports of secondary outcome data distributions. The CTU will alert RAs to any data that are missing or inaccurate. The linkage between personal and routine data and the individual participants will be stored separately and securely in a password-protected file.

#### Data security

Wherever possible, all personal and research data will be entered and stored only in electronic format. Where it is necessary to store personal or research data in hard copies, for example where there is no access to a laptop or where staff complete paper versions of a questionnaire, data will be stored at the designated NHS Trust base in a locked filing cabinet. Electronic copies of personal and study data will be stored on secure shared drives at each NHS site. All study data will be password-protected using a password known only to the study team. No personal or study data will be downloaded or stored on individual employee drives or desktops. Data will be entered onto the MACRO eCRF which is the electronic data management system and is Good Clinical Practice (GCP) and 21 CFR Part 11 compliant. Research data will be archived at each site, and centrally for all centrally collated electronic data and stored for a 10-year duration in line with sponsor policy. After the 10-year period, research data will be shredded, deleted or destroyed using confidential data destruction measures in place for each organisation. Audio recordings will be uploaded to the secure shared drive at each site and stored in an anonymised and encrypted form. The audio-recording will then be deleted from portable devices within 24 h of recording. Data will be stored confidentially and securely. Anonymised outcome data will be stored separately from personal information. All data will be stored in a password-protected format and any data that is transferred will be done securely and using encrypted zip files in for example, csv, Stata, SAS or SPSS format. Data will not be shared with anyone outside of the project team and organisations hosting the research.

### Confidentiality {27}

The project team will adhere to Good Clinical Practice standards, principles and policies for Data Protection, Security and Confidentiality, consistent with recommendations from current legislation, including The Caldicott Report (1997), the British Standard (ISO IEC 27002) for Information Security, the Data Protection Act, 1998, the Sussex Partnership NHS Foundation Trust Research Policy 2012, NHS Research Governance Framework 2005, HRA and research ethics approval processes. These principles relate to the need to protect personal data and guard against any unauthorised use, inform patients (and professionals) of its use and allow patients choice regarding how their personal data is disclosed or used. Participant personal data will be stored in a secure password-protected file at each study site. The drive will only be accessible to the RAs who are employed and working as part of the EIP clinical and research teams. In both hard and electronic versions, personal and study data will be kept separate. Study data will be identified using a participant identification number (ID). This ID will be linked to the participant’s name in a linked file. This file will be password-protected, with password known only to the study team.

### Plans for collection, laboratory evaluation and storage of biological specimens for genetic or molecular analysis in this trial/future use {33}

Not applicable. See above 26b there will be no biological specimens collected.

### Statistical methods

#### Statistical methods for primary and secondary outcomes {20a}

A full statistical analysis plan will be written and independently reviewed prior to unblinding and any analysis being undertaken.

##### RCT analysis

We will report all participant flow in line with the CONSORT 2010 Statement extension for cluster RCTs [80] showing withdrawal and loss to follow-up. Analyses will be conducted on an intention-to-treat (ITT) basis. Time to disengagement will be compared between trial groups using Cox regression with a gamma-distributed shared frailty to allow for the clustering by service. If this analysis fails to converge, we will employ fully parametric time-to-event regression analysis with shared frailty. Analyses will be conducted using Stata v16 or above (Stata Corporation, College Station, TX, USA). With a relatively small number of clusters per group, there is a risk that the type I error rate will be inflated—we will use a permutation test or similar approach in order to obtain a true significance level. Time to disengagement or the time beyond which observations are censored (due to drop-out or end of data collection) will be known for all participants. Secondary, quantitative outcome measures will be analysed using mixed regression analysis of all non-missing data (valid if outcomes are ‘missing at random’), with a random effect for service and a Kenward-Roger small-sample correction. We will investigate the sensitivity of our conclusions to the missing at random assumption by imputing outcome data under departures from this assumption. Secondary analyses will be conducted to investigate whether the intervention effect is mediated by adherence and context effects, as measured in the process evaluation.

We will adjust for measured service-level factors (e.g. variation in NICE interventions, deprivation) and individual-level factors (e.g. ethnicity, gender, duration of untreated psychosis) which could be important in predicting outcome: these will be finalised in the statistical analysis plan prior to locking the database and unblinding.

##### Economic analysis

The economic analysis will be composed of

1. A primary cost-effectiveness analysis conducted from a broad societal perspective including primary care, social services, police and criminal justice contacts, education and training services and occupational outcomes. This will examine patient outcomes (measured using HoNOS scores) alongside the incremental societal costs arising from the intervention over the 12-month trial follow-up period. This will include an assessment of the costs of investing in staff training in intervention methods, the cost of increased engagement with EIP and other NICE-recommended interventions, cumulative savings from reduced inpatient admissions and A&E contacts and the intervention impact on the cost of wider service contacts and outcomes (e.g. primary care, police and criminal justice systems, employment). Data will also be collected on utilisation of education and training services, though these activities will not be costed. The cost-effectiveness analysis will subsequently combine evidence on the cost implications of the EYE-2 intervention with health outcomes data (HoNOS scores) to evaluate whether EYE-2 was cost-saving (from a societal perspective) and equivalent or superior (to usual care) in terms of patient outcomes, or whether improved patient outcomes were achieved at greater overall cost over the follow-up period of the trial.

2. A secondary analysis of cost-effectiveness that takes a narrower NHS (commissioner and provider) perspective by combining health outcomes data with an examination of intervention impacts purely within mental health and other NHS services (e.g. psychiatric inpatient admissions and A&E attendance). We will also use HoNOS data to determine the mental health cluster (and therefore tariff) to which a service user would be allocated based on assessment of need at 12 months (e.g. a ‘step up’ or ‘step down’ service need). This will serve as a means to approximate the impact of the intervention on potential future commissioning resources based on payments tariffs linked to mental health cluster.

Service use measured through administrative and self-report data (via the AD-SUS) will be combined and costed using appropriate unit cost evidence either newly developed where necessary (if gaps in unit cost evidence exist) or from existing sources (e.g. Unit costs of Health and Social care, PSSRU; NHS Reference Costs). Employment outcomes (including absenteeism or employment gained or lost) will be valued using the ‘human capital’ approach (using occupational pay rates to value time spent in or out of paid or unpaid work). Estimated societal costs per trial participant will be examined in total and by service sector so that further insight into the distributional burden of costs by sector for this patient group can be gained.

The economic evaluation will quantify uncertainty in cost-effectiveness estimates due to second-order (sampling) uncertainty relating to costs and mental health outcomes. Where relevant, we will also explore, through the deterministic one-way sensitivity analysis, the sensitivity of conclusions reached to any key assumptions required.

##### Process evaluation analysis

The process evaluation will follow a mixed methods analysis approach. Questionnaire data will be used to produce scores for implementation (use of EYE-2 resources with service users) which will be analysed using mixed regression analysis of all non-missing data (valid if outcomes are ‘missing at random’), with a random effect for service and a Kenward-Roger small-sample correction to explore changes in scores over time, for implementation of the EYE-2 intervention through core mechanisms of coherence, cognitive participation, collective action and reflexive monitoring. Secondary analyses will be conducted to investigate whether the intervention effect is mediated by adherence and context. Outcomes across sites will be investigated in relation to turbulence (macrolevel stressors, complexity and changes within the NHS) over time. Qualitative interview data will explore implementation, barriers, facilitators, contextual effects and turbulence in the service (macro stressors, complexity, change) over time. Qualitative data will be analysed using constant comparative analysis [81] and interpreted in the light of Normalisation Process Theory.

### Interim analyses {21b}

Not applicable. There are no interim analyses planned.

### Methods for additional analyses (e.g. subgroup analyses) {20b}

Further details regarding analysis will be provided in the statistical analysis plan prior to commencing analysis.

### Methods in analysis to handle protocol non-adherence and any statistical methods to handle missing data {20c}

Further details regarding analysis will be provided in the statistical analysis plan.

### Plans to give access to the full protocol, participant-level data and statistical code {31c}

Plans will be made available at a later date.

## Oversight and monitoring

### Composition of the coordinating centre and trial steering committee {5d}

Sussex Partnership NHS Foundation Trust will be the sponsor. At each site, RAs, service user researcher, service users and carer will form a mini-team, supervised locally by the site lead, and centrally by the CI and CTU. Monthly project management meetings chaired by the CI and involving all co-applicants will manage day-to-day project management, ensure good communication between sites, receive monthly site reports on data collection, intervention delivery and progress and address problems. The trial steering committee (TSC) will comprise independent chair, clinical implementation academic, statistician, health economist and PPI member plus CTU lead and CI. It will meet 6-monthly, or more often if required, to provide overall trial supervision and independent advice, including review of project reports, protocols, amendments and adherence to protocols.

The CTU will oversee study conduct and ensure adherence to standard operating procedures (SOPs) and protocol. The trial manager will work with the study team to ensure that the study is managed in accordance with all regulations and governance frameworks. They will monitor recruitment and study conduct based on a risk adaptive approach using CTU SOPs and provide reports to the study team and oversight committees. The trial manager together with the CI will train staff in study requirements. Data entry will be quality controlled by the system as it is being entered (flagging up errors in real time). The data manager will monitor data collection and management in accordance with protocol, including design of data collection tools, undertaking data validation checks and writing the data management plan. They will work with sites to ensure all data entry is accurate and entered in a timely manner. They will provide data and information for oversight committees and undertake data cleaning prior to statistical analyses. All data will be archived in the Trial Master File and retained securely for a minimum of 5 years following completion and closure of the trial.

### Composition of the data monitoring committee, its role and reporting structure {21a}

An independent DMEC committee, including clinical chair, clinical academic and statistician will meet 6-monthly or more often if required, prior to the TSC. It will review trial data and serious adverse reactions and consider if for ethical or safety reasons the trial should end early. It is independent of the sponsor and there are no competing interests. The DMEC charter can be found on the study website at https://www.sussex.ac.uk/research/projects/eye-2/.

### Adverse event reporting and harms {22}

A trial-specific SOP has been written for the monitoring and reporting of adverse events in this trial. This trial is unusual in that it is a pragmatic cluster trial which includes the entire cohort of 20 EIP services in England for 1 year, followed up for a further year. It is therefore expected that there will be a very large number of serious adverse events (SAEs) due to the natural fluctuation in severity of psychosis experiences in this population, and the intervention itself is a comparatively low risk social, motivational and psychoeducation intervention. For this reason, it was decided to take a pragmatic approach to reporting of adverse events; this has been discussed with the trial management team and DMEC, and follows the approach used in a previous cluster RCT [82]. Clinicians and clinical link persons (team leader or clinical psychologist) in each team will raise to the research team any SAE that is deemed to be possibly, probably or definitely related to the trial, for further action. Seriousness is defined using standard criteria as (1) resulting in death; (2) is life threatening (only including self-harm or suicide ideation or suicide attempt requiring hospitalisation or serious threat or act of harm to others); (3) requires inpatient hospitalisation or prolongation of existing hospitalisation (only in the case of suicide ideation grade 4 or suicide attempt grade 4 or 5 as according to the Common Terminology Criteria for Adverse Events (CTCAE) version 5.0 November 2017, or serious threat or act of harm to others requiring hospitalisation; (4) resulting in persistent or significant disability or incapacity. Relatedness criteria include (1) distress caused by contents of booklet or website; (2) distress or fatigue caused by answering questionnaires or taking part in interviews; (3) disappointment at being allocated to the control group; (4) distress related to the involvement with or breach of confidentiality related to involvement of a non-standard member of the social network such as friend or other non-family member; (5) distress triggered by attendance at an EYE-2 social group; (6) distress caused by concern about data security in the trial (routine data/posts on the forum); (7) distress caused by a response to a post on the forum or other forum content; (8) other as deemed by site. Events that are not serious or not related to the trial (adverse event (AE), serious adverse event (SAE), unexpected adverse event (UAE), adverse reaction (AR)) will require no further action. Any event rated as at least possibly serious and possibly related will be reported immediately to the EIP RA who will gather additional information and complete a draft serious adverse reaction (SAR) form on the same day and email this to the CTU and site PI. The site PI will reassess causality and expectedness and the CTU will send the information to the CI. Participants will be followed up until clinical recovery is complete, or until the event has resolved, and information reported on the final SAR report. In the absence of the PI, the form should be completed and signed by another trained member of the site trial team who is named on the delegation log (as designated by local PI). The PI should subsequently check the SAR form, make changes as appropriate, sign and then send to the Brighton and Sussex CTU as soon as possible. The patient must be identified by trial number. The patient’s name should not be used on any correspondence. This final SAR report is then graded SAR or suspected unexpected serious adverse reaction (SUSAR) on the basis of expectedness judged by the PI and CI. The CTU will notify the research ethics committee of SUSARs as per the conditions of the favourable opinion and according to CTUSOP018 within 15 calendar days of the CTU first being notified of the event. The CI and independent clinical reviewer will assess any possible SARs. Events recorded as serious, related and expected (SAR) will be submitted to the DMEC and sponsor as required. Any events rated as also unexpected (SUSAR) will also be reported to the REC within 15 days of first notice. This may involve the DMEC members being unblinded to the trial group or seeking further data. If there are any ethical or safety reasons why the trial should be prematurely ended, they will advise the TSC accordingly.

### Frequency and plans for auditing trial conduct {23}

Auditing of trial conduct will be conducted regularly by the CTU, including monitoring of site file completion and conduct at each site, 6-monthly by the TSC and DMEC, and annually by the sponsor.

### Plans for communicating important protocol amendments to relevant parties (e.g. trial participants, ethical committees) {25}

All protocol amendments will be submitted for HRA/ethics approval, and revised protocols provided to the funder. All approved amendments will be communicated by the CTU to all site PIs and local governance teams prior to implementation.

### Dissemination plans {31a}

The study website (www.sussex.ac.uk/research/projects/eye-2) will be a focal point for disseminating outputs, through newsletters, presentations, high-impact peer-reviewed academic and service user publications and a tailored VLOG to service users, relatives, teams, regional and national networks. Participants will be able to provide comments and suggestions for dissemination. All national services will be invited to a results launch event, which will be recorded and added to the study website, along with other outputs. The implementation toolkit will be formed into a series of implementation packages, tailored to different contexts, including training, manuals, checklists, website, booklets, schools pack and social involvement protocols. These will be made available to clinicians, managers and services to support delivery in the NHS. A broader package of learning, relating to implementation in youth and mental health services, will be made available for other youth and psychosis services (NHS and non-statutory). The commissioning guide, developed with our GP commissioner, will be provided for commissioning purposes. Our collaboration with NHS England EIP lead (JN) will allow us to adapt approaches and materials during the study, and release these to support and guide future NHS England targets. We will present our findings to the public, participants, services and academic audiences through the Sussex Psychosis Research Interest Group, and other site-specific and local feedback events, national and international conferences. We will draw on our national collaborations, and regional links, so that if effective, we can readily disseminate the outcomes of this study, and guidance for implementation to all EIP teams in England, alongside the manuals, and commissioner guidance. A series of Tweet chats involving national and international colleagues, offered to services throughout the UK, will support further implementation planning. Researchers, clinicians and services will thus be kept informed and able to use new information regarding (i) the effectiveness and cost-effectiveness of the intervention; (ii) the engagement needs of ethnic minority EIP populations and (iii) variations in implementation and outcomes based on NHS service context, turbulence, macro and micro stressors. Service users and families will thus, in a timely manner, receive engagement-focussed services, supported by ‘myth-busting’ resources that address their personal goals and needs.

EIP services throughout the UK will be supported to implement the EYE intervention. The results of the study, if effective and cost-effective, will be widely disseminated through our network of regional and national channels, to clinicians, services, trusts and CCGs, supported by NHS England. We will offer a set of training and implementation packages tailored to different contexts and services, including manuals, resources and commissioner guides. The Normalisation Process Theory framework will enable us to lay out specific changes that will be required in terms of roles and responsibilities, beliefs, behaviours, relationships, processes and structures to deliver the EYE-2 approach at an individual, social network, service and NHS trust level. This will enable a real and meaningful change in how individuals work and services are delivered, based on core EYE intervention principles.

## Discussion

Over the past 20 years, significant gains have been made nationally and internationally in ensuring that early detection and intervention services are available for young people who develop psychosis for the first time, and their families. Substantial recent investment has helped to reduce waiting times to 2 weeks from referral to allocation to a care coordinator, for the majority of cases, as well as ensuring that EIP service provision is equipped with a full range of NICE-recommended treatment options. Yet approximately 25% of young people disengage prematurely without receiving the full benefit of these services and at significant potential cost to their mental health. Whilst strategies are in place to support engagement with an assessment process, there are no interventions aimed at improving engagement in the longer term, and as young people begin to disengage.

The EYE-2 trial is the first to evaluate a specific engagement-focussed intervention for young people with psychosis. The pragmatic cluster RCT design will evaluate effectiveness and cost-effectiveness. The use of NHS England mandated outcomes serves to strengthen follow-up data completion, across the whole sample, including for those who are disengaging. The implementation process evaluation, delivery through routine clinical service structures and provision of an implementation tool kit, training programme and resources will ensure that the intervention is highly scalable and will distinguish it from standard EIP care. Staff responses to the process evaluation and booster training will ensure that the intervention can be adapted to suit a variety of service structures, clinician needs and preferences, both within and beyond EIP.

Patient and public involvement (PPI) has been integral to the conduct, intervention and resource development in the original EYE project and PPI continues to be integral to the current study design. PPI will be led from the McPin Foundation (specialists in mental health service user research). PPI activities will include (i) contribution to steering group and study meetings; (ii) reviewing ethics, recruitment and advertising materials; (iii) supporting training delivery at each site; (iv) supporting the delivery of the social groups at each intervention site; (v) co-facilitating the lived experience group at each site; (vi) contributing to the dissemination plan. In each site, the senior PPI lead, 2 service users and a parent will be involved in the local EYE-2 staff training and EYE-2 social group programme. The entire PPI team will contribute to articles, VLOGS and study newsletters. All of this previous and planned work will ensure that the intervention and materials remain highly valued by clinicians, service users and their families and that service users continue to feel they have more choices, enhanced goals, increased hope, trust and quality of life.

## Trial status

The protocol is version 4: Dated 3rd April 2020. Service user identification (recruitment) began on 13th May 2019 and is expected to complete during July 2020.

## Supplementary Information

**Additional file 1.** CONSORT 2010 Flow Diagram.

## Abbreviations

EYE: Early Youth Engagement
EIP: Early Intervention Service
NHS: National Health Service
RCT: Randomised controlled trial
UK: United Kingdom
NICE: National Institute of Health and Care Excellence
HoNOS: Health of the Nation Outcome Scale
QPR: Process of Recovery Questionnaire
GP: General practitioner
NPT: Normalisation Process Theory
SPIRIT: Standard Protocol Items: Recommendations for Interventional Trials
FEP: First episode of psychosis
RA: Research assistant
PPI: Patient and Public Involvement
AR: Adverse reaction
SAR: Serious adverse reaction
AD-SUS: Adult Service Use Schedule
A&E: Accident and emergency
PI: Principal investigator
CI: Chief investigator
CTU: Clinical trials unit
SNOMED-CT: Systematized Nomenclature of Medicine Clinical Terms
eCRF: Electronic Case Record Form
GCP: Good Clinical Practice
ID: Identification number
TSC: Trial Steering Committee
SOP: Standard operating procedure
DMEC: Data Monitoring and Ethics Committee
SAE: Serious adverse event
AE: Adverse event
UAE: Unexpected adverse event
CTCAE: Common Terminology Criteria for Adverse Events
REC: Research ethics committee
HRA: Health Research Authority
VLOG: Video Log
CCG: Clinical Commissioning Group
