# Supplementary material for: The Early Youth Engagement in first episode psychosis (EYE-2) study: pragmatic cluster randomised controlled trial of implementation, effectiveness and cost-effectiveness of a team-based motivational engagement intervention to improve engagement
Source: Trials. 2021 Apr 12;22:272. doi: 10.1186/s13063-021-05105-y (PMC8042707; doi:10.1186/s13063-021-05105-y)
Supplement: Supplementary file 1 — Additional file 1. CONSORT 2010 Flow Diagram. [file 13063_2021_5105_MOESM1_ESM.doc]

**CONSORT 2010 Flow Diagram**

**Analysis**

**Enrollment of Teams**

**Eligibility screening**

**Baseline**

**6 Month follow-up**

**12 Month follow-up**

**Allocation**

**18 Month follow-up**

**24 Month follow-up**

Teams identified that met inclusion criteria (n= )

Analysed (n= )
♦ Excluded from analysis (give reason) (n= )

- Withdrawn (n = )*
- Missing at baseline (give reason) (n= )
- Data collected (n = )

Analysed (n= )
♦ Excluded from analysis (give reason) (n= )

Teams randomized (n= )

(n= )

Service users screened for eligibility (n =)

Service users excluded with reasons

- Not first episode psychosis (n =)
- Aged over 35 years (n = )
- Aged under 14 years (n = )

Service users included in cohort (n = )

Service users screened for eligibility (n =)

Service users excluded with reasons

- Not first episode psychosis (n =)
- Aged over 35 years (n = )
- Aged under 14 years (n = )

Service users included in cohort (n = )

Teams lost (n= _)

- Withdrawn (n = )*
- Missing at baseline (give reason) (n= )
- Data collected (n = )
- Withdrawn (n = )*
- Lost to follow-up (give reason) (n= )
- Data collected (n = )
- Withdrawn (n = )*
- Lost to follow-up (give reason) (n= )
- Data collected (n = )
- Withdrawn (n = )*
- Lost to follow-up (give reason) (n= )
- Data collected (n = )
- Withdrawn (n = )*
- Lost to follow-up (give reason) (n= )
- Data collected (n = )

Allocated to EYE-2 + EIP intervention

(Teams: n= )

Allocated to EIP intervention

(Teams: n= )

- Withdrawn (n = )*
- Lost to follow-up (give reason) (n= )
- Data collected (n = ) Censored (n= )
- Withdrawn (n = )*
- Lost to follow-up (give reason) (n= )
- Data collected (n = ) Censored (n= )

Teams lost (n= )

- Withdrawn (n = )*
- Lost to follow-up (give reason) (n= )
- Data collected (n = ) Censored (n= )
- Withdrawn (n = )*
- Lost to follow-up (give reason) (n= )
- Data collected (n = ) Censored (n= )
